# Bacterial Extracellular Vesicles: An Emerging Concern in the Field of Environmental Contamination—Characterization, Impacts, and Response Strategies

**DOI:** 10.3390/microorganisms13102413

**Published:** 2025-10-21

**Authors:** Yu Fu, Yaoqiang Shi, Zhijian Zhou, Yafei Huang, Yuzhu Song, Chao Li

**Affiliations:** 1 Research Center of Molecular Medicine of Yunnan Province, Faculty of Life Science and Technology, Kunming University of Science and Technology, Kunming 650500, China; fuyu021018@163.com (Y.F.); yaoqiangshi94@gmail.com (Y.S.); 13535108596@163.com (Z.Z.); 20241118006@stu.kust.edu.cn (Y.H.); 2 Institute of Basic and Clinical Medicine, The First People’s Hospital of Yunnan, The Affiliated Hospital of Kunming University of Science and Technology, Kunming 650032, China

**Keywords:** bacterial extracellular vesicles, environmental pollutants, resistance genes, virulence factors, transmission

## Abstract

Bacterial extracellular vesicles (BEVs) are nanoscale membrane-bound structures secreted by prokaryotic cells and have recently gained considerable attention in environmental pollution research. By encapsulating virulence factors and antibiotic resistance genes (ARGs), BEVs can persist in aquatic, soil, and sedimentary environments, facilitating interspecies gene transfer, aggravating microbial contamination, and ultimately posing risks to ecosystem stability and human health. This review provides a comprehensive overview of BEVs’ formation mechanisms, structural composition, and spatial distribution. Particular attention is given to the environmental implications of BEVs, including their roles in mediating horizontal ARG transfer, delivering virulence genes and amplifying pathogenicity, and their emerging potential as environmental bioindicators, despite current analytical limitations in complex matrices. Nevertheless, three major research gaps remain: (i) the molecular mechanisms underlying BEV interactions with heavy metals and microplastics are poorly understood; (ii) field-based quantification and distribution data are still limited; and (iii) effective, targeted strategies for BEV removal or inactivation are lacking. Addressing these challenges will not only enhance our understanding of BEV-mediated environmental risks but also inform the development of advanced detection methods and remediation approaches for BEV-associated pollution.

## 1. Introduction

**Figure 1:**
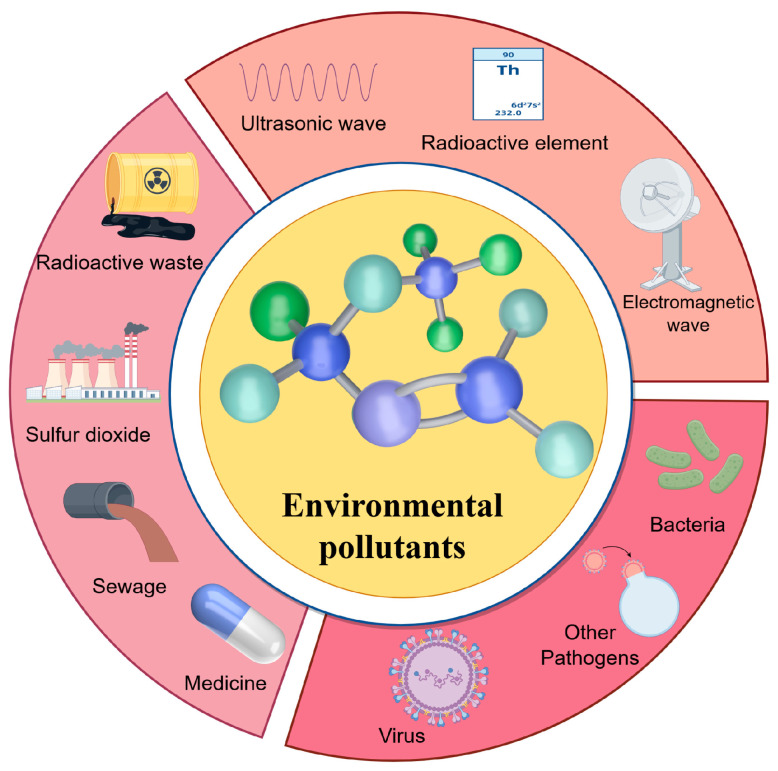
Classification of common environmental pollutants. Environmental pollutants are generally classified into three categories according to their intrinsic properties: physical pollutants, including ultrasonic waves, radioactive elements, and electromagnetic radiation; chemical pollutants, such as radioactive waste, sulfur dioxide, and sewage; and biological pollutants, comprising viruses and bacteria.

The degradation of the human ecological environment has emerged as a pivotal focus in environmental science, drawing significant global attention in the context of accelerating industrialization. This concern arises from the unintended consequences of human activities that prioritize material prosperity over sustainable practices, resulting in the release of over 7 million chemical substances into natural systems. While these substances facilitate modern life, they also inflict direct and indirect harm on the environment [1]. Environmental pollutants are defined as substances that disrupt the composition and functionality of air, water, and soil. They include both anthropogenic byproducts, such as sulfur dioxide from coal-fired power generation and heavy metals like mercury, cadmium, and lead from waste batteries, as well as natural emissions, including volcanic gases and particulates [2]. As shown in Figure 1, these contaminants can be taxonomically categorized into physical, chemical, and biological classes [3], with the latter being notable for their potential to harbor antibiotic resistance genes (ARGs) and virulence determinants. In recent years, the horizontal transfer of ARGs via bacterial extracellular vesicles (BEVs) has been emerged as a novel mechanism driving microbial adaptation and the propagation of pathogenicity [4,5,6,7]. This process warrants in-depth investigation, as ARGs confer antibiotic tolerance through persistent environmental dissemination, enabling interspecies genetic exchange and bacterial evolutionary divergence [8]. Structurally, BEVs are spherical nanovesicles (20–400 nm) spontaneously released by prokaryotes. They are delimited by a phospholipid bilayer derived from the parental cell envelope and are laden with diverse biomolecules, including membrane proteins, periplasmic factors, and cytoplasmic effectors [9,10]. Initially characterized in *Escherichia coli*, these vesicles exhibit structural stability and prolonged environmental persistence, which facilitate intercellular communication, stress responses, quorum sensing, and the transfer of virulence traits [8]. Functionally, BEVs serve as multifunctional mediators in microbial ecology: they enable horizontal gene transfer (HGT) of ARGs, facilitate nutrient acquisition, provide defense against predation, and mediate electron shuttle processes across heterogeneous ecosystems [11]. Reported in all prokaryotic and eukaryotic kingdoms as integral components of cellular physiology [12,13], BEVs have been systematically characterized in laboratory cultures of aquatic bacteria spanning latitudinal gradients (from polar Antarctic isolates to mid/high-latitude marine strains) with scanning electron microscopy confirming their presence in coastal and open-ocean seawater samples [14,15,16]. Freshwater bacterial cultures further demonstrate their ecological ubiquity, highlighting BEVs as conserved constituents of both marine and freshwater habitats [17,18,19]. Collectively, these findings underscore the global distribution of BEVs and their critical roles in shaping microbial community dynamics and environmental pollutant cycles.

**Figure 2:**
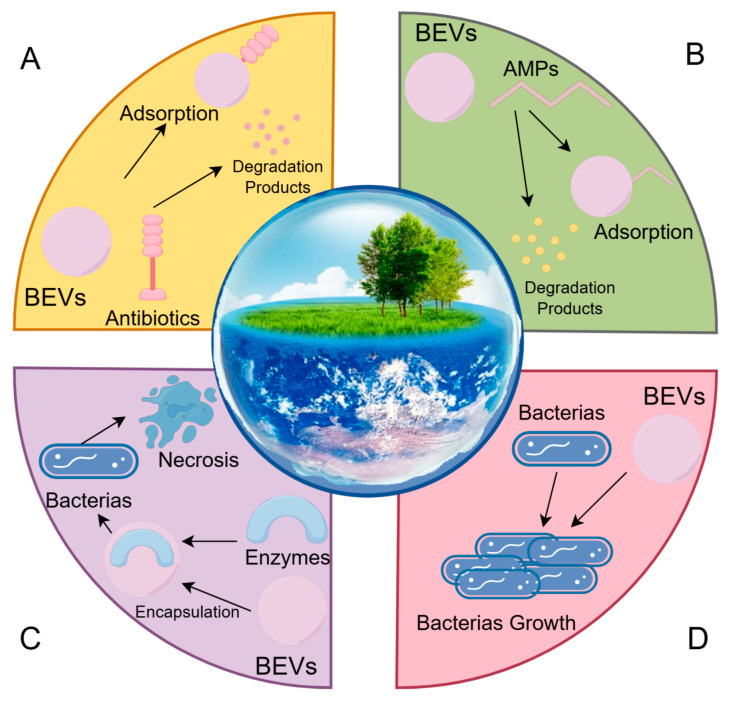
Roles of BEVs in Ecosystems. (**A**) BEVs can bind to and degrade antibiotics, thereby functionally inactivating them and protecting bacteria from antibiotic-mediated killing. (**B**) BEVs are capable of adsorbing and even degrading antimicrobial peptides, thus neutralizing their bactericidal activity. (**C**) BEVs can encapsulate and deliver peptidoglycan hydrolases to target bacteria, leading to cell wall degradation and subsequent bacterial lysis. (**D**) BEVs facilitate the horizontal transfer of ARGs and virulence factors, influencing bacterial survival and evolution.

The secretion of BEVs represents an adaptive defense strategy employed by bacteria in response to antibiotic or bacteriostatic stress. This mechanism enables the sequestration of antimicrobial agents and modulation of microbial interactions (Figure 2). Mechanistic studies have demonstrated that BEVs isolated from *Staphylococcus aureus* cultures effectively bind to daptomycin (a membrane-targeting antibiotic), thereby shielding the parental bacteria from drug-mediated membrane disruption. In this context, the production of antibiotic-induced BEVs acts as a decoy mechanism that enhances bacterial survival [20]. Manning et al. reported that polymyxin B and visfatin (both peptide antibiotics) trigger the release of BEVs from *E. coli*, where these vesicles adsorb and neutralize antimicrobial peptides, thereby abrogating their bactericidal activity [21]. Beyond direct antibiotic sequestration, BEVs may also carry antibiotic hydrolases (such as β-lactamases) to enzymatically degrade antibiotics [22]. This vesicle-mediated resistance phenotype not only undermines antibiotic efficacy but also facilitates the persistent dissemination of ARGs in environmental niches, promoting interspecies genetic exchange and evolutionary divergence of bacterial populations [23]. Furthermore, BEVs serve as tools for interbacterial warfare: *Pseudomonas aeruginosa*-derived BEVs deliver cytoplasmic hydrolases capable of degrading peptidoglycan, thereby killing both Gram-negative and Gram-positive competitors [24]. Similarly, *S. aureus* BEVs enriched with N-acetylcytidylyl-L-alanine amidase compromise neighboring bacteria through the hydrolysis of peptidoglycan [25]. These lytic activities, coupled with the transfer of ARGs and virulence determinants disrupt microbial community homeostasis [26]. Such disruptions promote the dominance of specific bacterial lineages, accelerating biofilm formation and colonization of ecological niches. These processes pose significant risks to the balance of environmental microbiota and human health by enhancing pathogenicity and treatment resistance.

ARGs have emerged as a novel class of environmental contaminants [27], distinguished from conventional pollutants by their capacity to persist, replicate, and spread horizontally across microbial communities. Once acquired by pathogenic bacteria, ARGs undermine the efficacy of antibiotic therapies, thus posing a significant threat to public health through the reduction in available antimicrobial treatment options [28]. Encapsulated within a phospholipid bilayer derived from the bacterial cell envelope, BEVs exhibit structural integrity that enables the stable transport of cytotoxic proteins, virulence determinants, and other bioactive molecules [29,30]. This membrane-bound architecture not only protects encapsulated cargo from environmental degradation but also facilitates intercellular signaling and molecular exchange, positioning BEVs as critical mediators of bacterial–bacterial and bacterial–host interactions [31]. At the microbial community level, BEVs serve as nanoscale communication hubs, enabling bacteria to modulate their microenvironment through intercellular signaling and HGT. These interactions facilitate coordinated bacterial behaviors (such as biofilm formation and quorum sensing), while enhancing genomic plasticity via the dissemination of ARGs and mobile genetic elements [32,33,34]. In the context of host–pathogen dynamics, BEVs-mediated interactions elicit diverse cellular responses, ranging from non-immunogenic tolerance to pro-inflammatory activation or cytotoxic injury. These outcomes are contingent on multiple factors, including the taxonomic identity of the bacterial donor, the phenotypic characteristics of target cells, and the dose-dependent abundance of vesicles [35].

In recent decades, microbiological perspectives have emerged as critical for re-evaluating pollution dynamics, with BEVs—nanoscale mediators of microbe–environment interactions —expanding the frontiers of environmental microbiology and pollution ecology. By characterizing BEVs as novel bioactive contaminants, researchers can enhance mechanistic understanding of pollutant classification and systemic impacts, thereby informing the development of precision-targeted environmental quality standards and monitoring frameworks. This review first systematically examines the formation mechanisms, structural composition, and environmental distribution characteristics of BEVs, considering their widespread distribution in the environment and their significant role in the transmission of pollutants. It then analyzes the mechanisms by which BEVs are associated with environmental pollution, and finally discusses current research limitations and future directions. This comprehensive analysis aims to provide theoretical support for the risk management and control of BEV-related pollution.

## 2. Characteristics of BEVs

### 2.1. Formation Mechanisms

**Figure 3:**
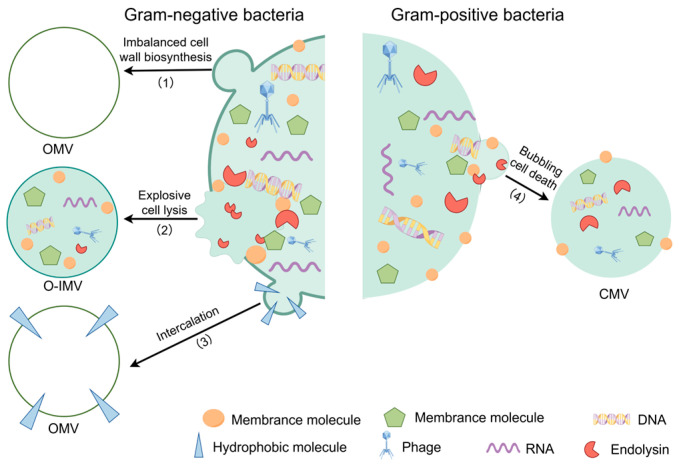
Mechanisms of BEVs biogenesis. In Gram-negative bacteria, BEVs (specifically OMVs) are formed through: (1) imbalanced cell wall biosynthesis, causing outer membrane blebbing; (2) explosive cell lysis, producing vesicles from both membrane layers (O-IMVs); and (3) the insertion of hydrophobic molecules. In Gram-positive bacteria, CMVs are generated via bubbling cell death (4).

BEVs are widely distributed across the prokaryotic kingdom [25,36,37,38], with both Gram-negative and Gram-positive bacteria generating distinct vesicle subtypes through specialized biogenesis pathways. This compositional diversity arises from lineage-specific production mechanisms, highlighting the functional versatility of these nanovesicles in microbial ecology. Gram-negative bacteria secrete outer membrane vesicles (OMVs), which are derived from the asymmetric outer membrane of the cell envelope. These OMVs encapsulate a cargo of periplasmic enzymes, cytoplasmic proteins, and nucleic acids, typically measuring between 20 and 250 nm in diameter [9] (Figure 3). In contrast, Gram-positive bacteria produce cytoplasmic membrane vesicles (CMVs) that originate from the phospholipid bilayer of the cytoplasmic membrane and carry cytoplasmic lysate components within structures ranging from 20 to 400 nm [9]. Disruption of the peptidoglycan layer, a structural polymer unique to bacterial cell walls, has been implicated in plasma membrane protrusion, which drives OMV biogenesis and may yield outer-inner membrane vesicles (O-IMVs) that incorporate both membrane bilayers of Gram-negative bacteria [39,40]. Although species-specific differences in BEV biogenesis remain an active area of research, two evolutionarily conserved pathways have been identified across bacterial phyla. The first is membrane blistering, which occurs spontaneously during the exponential growth phase. This process is typically triggered by an imbalance in cell wall biosynthesis or by the intercalation of hydrophobic molecules into the membrane. These perturbations induce lateral lipid phase separation, resulting in membrane protrusion and the subsequent budding of vesicles into the extracellular space [41]. The second pathway is explosive cell lysis, which is mediated by endolysins—phage-encoded enzymes that degrade peptidoglycan, leading to osmotic rupture of the bacterial cell. This mechanism releases both single-membrane BEVs and O-IMVs in Gram-negative species [42,43,44,45,46]. This lytic pathway is particularly significant for generating vesicles with a dual-membrane architecture, although its contribution to the production of single-membrane BEVs is also well-documented [46]. Environmental cues play a pivotal role in modulating BEV output, with parameters such as pH [47], oxygen tension [48], nutrient availability [49,50], temperature [51], and antibiotic exposure [22,52] serving as key regulators. For example, acidic pH alters the outer membrane lipid composition in Gram-negative bacteria, promoting OMV formation through membrane destabilization [47]. In *Microcystis aeruginosa*, oxygen-deprived conditions induce an SOS response via disrupted denitrification pathways, triggering differential production of BEV subtypes [48]. These adaptive responses underscore the ability of bacteria to upregulate or downregulate vesicle output in response to environmental fluctuations, thereby ensuring survival through modulation of intercellular communication, nutrient acquisition, and stress tolerance.

### 2.2. Composition and Structural Characteristics

The architecture of the bacterial cell envelope comprising cell wall polymers and membrane bilayers, dictates the biogenesis and release of BEVs. This results in significant interspecies and intraspecific variability in vesicle structure and cargo composition [53,54]. Gram-negative BEVs, exemplified by OMVs, encapsulate a diverse array of components, including lipopolysaccharides (LPS), membrane-associated proteins, lipids, and genetic material, with composition contingent upon the bacterial physiological state [55,56,57]. Biochemical analyses, such as sodium dodecyl sulfate–polyacrylamide gel electrophoresis, protein staining, and Western blotting, have identified conserved components, including outer membrane proteins (Omps) such as OmpA, OmpC, and OmpF, as well as periplasmic enzymes and virulence determinants (adhesins, invasins, and hydrolytic enzymes) in OMVs derived from Gram-negative pathogens [58,59,60,61,62]. In contrast, the first Gram-positive BEVs isolated from *S. aureus* and *Bacillus subtilis* culture supernatants in 2009, exhibit spherical, bilayered structures ranging from 20 to 100 nm in diameter, enriched with surface-exposed virulence factors [25]. Proteomic profiling reveals these CMVs carry over 90 vesicular proteins, including β-lactamases, coagulases, hemolysins, IgG-binding protein SbI, and N-acetylcytosyl-L-alanine amidase—enzymes critical for immune evasion and bacterial competition [37,63,64,65,66,67]. The genetic cargo within BEVs encompasses both luminal and surface-associated nucleic acids, with luminal DNA showing resistance to DNase digestion, a characteristic that distinguishes vesicle-associated DNA from free extracellular DNA [55]. Beyond DNA, BEVs transport microRNAs, mRNAs, and noncoding RNAs, facilitating intercellular communication processes such as antigen presentation [68] and iron homeostasis. *Mycobacterium*-derived BEVs, which carry mycobactin-type siderophores, efficiently deliver iron to cognate *Corynebacteriaceae* bacteria, highlighting their role in nutrient acquisition [69]. Pathogenic BEVs frequently exhibit cargo-specific virulence amplification; certain toxins and virulence factors, such as β-lactamases in *S. aureus* CMVs, are preferentially enriched in vesicles or exclusively localized to their membranes, thereby enabling targeted delivery to host cells or competing microbes [37,70,71,72,73]. For example, *S. aureus* BEVs containing DNA and small RNAs activate pattern-recognition receptors in host epithelial cells, leading to the induction of cytokine and chemokine expression that promotes inflammation and tissue invasion [37,74,75,76]. The functional versatility of Gram-positive vesicles is intrinsically linked to their cargo packaging mechanisms during biogenesis, with toxins, siderophores, and antibiotic resistance proteins collectively contributing to their roles in pathogenesis and environmental persistence [70,71,72,73,77].

### 2.3. Distribution

Current research on the environmental distribution of BEVs has documented their presence across diverse ecosystems, including glacial ice [78], atmospheric dust [79], seawater [78], geothermal hot springs [80], fecal matrices [81], and soil microenvironments [82,83]. Municipal wastewater treatment facilities, recognized as critical urban reservoirs for ARGs, were among the first urban environments where BEVs were visualized in biofilm matrices via transmission electron microscopy [84]. Qin Y et al. [85] characterized a taxonomically diverse array of BEVs in indoor dust samples, identifying 241 ARG subtypes conferring resistance to 16 commonly used antibiotics. Among these, *EF-Tu*, *rpoB*, and *parC* were the most prevalent, with relative abundances of 7.27%, 5.82%, and 5.22%, respectively, indicating a notable enrichment for multidrug-resistant determinants. This underscores their role in propagating antimicrobial resistance in human-associated habitats. These dust-borne BEVs, as components of particulate environmental pollutants, have been linked to inflammatory responses in the human respiratory tract [86,87]. In marine systems, seawater derived BEVs carry both DNA fragments (≥3000 bp) and RNA fragments, often encoding functional gene clusters [88]. Biller et al. [15] provided seminal evidence of 100 nm diameter BEVs released by marine cyanobacteria, demonstrating their ecological significance as vectors for nutrient exchange and genetic material transport in oligotrophic oceanic environments. Research on *Chlorella protothecoides*, a dominant photoautotrophic *cyanobacterium* found in low-trophic seas, reveal the constitutive secretion of abundant BEVs, highlighting their role in microbial carbon and nitrogen cycling [15]. Extreme environments, such as geothermal hot springs, are inhabited by hyperthermophilic and acidophilic *archaea* that employ the endosomal sorting complex required for transport machinery to produce BEVs ranging from 77 to 182 nm in size [81]. This evolutionarily conserved pathway facilitates HGT and metabolic cooperation under harsh conditions, highlighting BEVs as key facilitators of extremophile adaptation. In matrices derived from animals, fecal BEVs have been associated with systemic inflammatory responses that resemble septicemia, with studies linking their presence to interactions between hosts and microbiota [89,90,91]. Laboratory cultures of soil bacteria further confirm the capacity for BEV biogenesis in liquid media, suggesting that constitutive vesicle release occurs in natural terrestrial habitats [92,93].

Beyond environmental niches, BEVs have been identified in various animal biological fluids, including intestinal lumen contents, amniotic fluid, placental tissues, milk, and organ interstitial fluids. These vesicles are secreted by resident microbiota and shed into circulatory (serum), urinary, and salivary systems [93,94,95,96]. The continuous secretion of phenotypically identical or distinct BEVs by different bacterial strains across diverse habitats reflects a conserved adaptive strategy, enabling robust intercellular communication and stress tolerance via vesicle-mediated cargo delivery. Collectively, the ubiquitous presence of BEVs in diverse ecosystems underscores their structural stability, environmental persistence, and functional versatility in mediating microbial interactions across scales-from molecular signaling in extreme environments to modulating human health in urban settings. The differences in the distribution of BEVs across various environments are primarily attributed to the regulation of bacterial vesiculation rates by environmental factors, such as antibiotic concentration and pH. This distribution characteristic directly influences the spatial range of pollutants. The following section further analyzes the specific relationship between BEVs and environmental pollution.

## 3. Linkages Between BEVs and Environmental Pollutants

### 3.1. Mediating Horizontal Transfer of ARGs

BEVs serve as robust secretion nanosystems for the targeted delivery of biomolecules, including ARGs to surrounding microbes, host cells, and environmental matrices, thereby playing a pivotal role in the horizontal dissemination of ARGs [97]. Classical HGT pathways—conjugation, transformation, and transduction—operate under specific environmental conditions, which has led to ongoing research into novel transfer mechanisms. Soler et al. [98] formally proposed vesicle-mediated gene transfer as the fourth mode of HGT, emphasizing BEVs as nanoscale vectors that encapsulate and deliver plasmid or genomic DNA, thus facilitating the transport of condensed nucleic acids within and among bacterial taxa. The genetic material contained within BEVs is protected from nuclease degradation, in contrast to free extracellular DNA, and exhibits significant diversity within aquatic ecosystems, highlighting their potential to promote cross-species gene flow [99,100]. Notably, seminal studies have shown that *Thermus*-derived BEVs, when stored with nucleases at 4 °C for two years, maintained functional genetic material, thereby demonstrating their capacity as long-lived vehicles for HGT [101]. Seawater-isolated BEVs are enriched with DNA transposons further supporting their hypothesized role as key carriers of mobile genetic factors in marine environments [15,97]. Functional characterization of BEV-associated ARGs reveals the presence of plasmid-encoded resistance determinants across diverse taxa: *E. coli* MG1655 BEVs harbor colistin and melittin resistance genes [102]; *Aeromonas vivax* and *Enterobacter cloacae* BEVs carry the broad-host-range plasmid pLC2017, which encodes kanamycin resistance [103]; and *A. baumannii* BEVs encapsulate the OXA-24 carbapenemase gene, conferring resistance to penicillins, carbapenems, and cefepime [104]. While early research primarily focused on Gram-negative bacteria, Wang et al. [105] pioneered studies in Gram-positive systems, demonstrating for the first time that BEVs mediate ARG transfer in *enterococci*. Critically, vesicle-transformed bacteria that retain ARGs can further propagate these genes via secondary BEV secretion, establishing a self-sustaining cycle of resistance gene dissemination.

Factors governing the horizontal transfer of ARGs mediated by BEVs can be categorized into intracellular physiological determinants and extracellular environmental stimuli [106]. Extracellular factors including pH, oxygen tension, nutrient availability, temperature, and antibiotic exposure, are well documented modulators of BEV biogenesis, with each parameter influencing vesicle secretion rates and cargo composition. For instance, acidic pH or antibiotic stress often upregulates the release of BEVs in Gram-negative bacteria, facilitating the encapsulation and dissemination of ARGs [22,52]. In contrast, intracellular determinants, such as the taxonomic identities of donor and recipient bacteria, the copy number and structural characteristics of plasmids, and specific ARG subtypes, govern the efficiency of genetic cargo loading and intercellular transfer [6,107]. These intracellular factors are hypothesized to exert context-dependent effects on the propagation of ARGs; however, their mechanistic interplay with extracellular cues remains poorly characterized. While extracellular stimuli typically promote the spread of ARGs mediated by BEVs by enhancing vesicle production, the regulatory landscape of intracellular factors is more nuanced and understudied. For instance, the compatibility between donor plasmid backbones and recipient bacterial machinery may dictate whether encapsulated ARGs are functionally expressed in target cells, a process influenced by plasmid size, replication origin, and adaptive gene cassettes [107]. These complexities underscore the necessity for systematic investigations into how bacterial genotypes and physiological states influence the biogenesis of BEVs and the selection of ARGs they carry. The ecological and clinical implications of ARG transfer mediated by BEVs are substantial: by enhancing bacterial tolerance to antibiotics, these vesicles compromise the efficacy of antimicrobial therapies, thereby facilitating the persistent dissemination of ARGs within environmental niches and promoting genetic exchange across species [28]. This process not only accelerates the evolution of multidrug-resistant bacterial lineages but also disrupts the homeostasis of microbial communities, posing significant risks to ecosystem resilience and human health through compromised infection treatment and increased pathogen virulence.

### 3.2. Transmission of Virulence Genes

Encapsulated within a phospholipid bilayer derived from the bacterial cell envelope, BEVs exhibit structural integrity that protects their internal cargo from environmental degradation [29,30], including cytotoxins, virulence determinants, and bioactive molecules. This membrane-bound architecture enables BEVs to act as efficient nanovehicles for intercellular trafficking, facilitating the targeted delivery of virulence factors and modulating host–pathogen interactions [31]. Experimental evidence highlights the superior potency of vesicle-mediated toxicity compared to free toxins: *E. coli*-derived BEVs carrying the pore-forming cytotoxin Cly-A exhibit a 10-fold increase in erythrocyte lysis activity compared to equimolar concentrations of purified Cly-A protein. This enhanced activity is attributed to the stability of the protected cargo and the localized concentration of the toxin at target membranes [108]. Furthermore, in vivo studies demonstrate that BEVs induce robust immune responses through receptor-mediated recognition; intraperitoneal administration of *E. coli* BEVs in mice triggers TLR4- and ICAM-1-dependent neutrophil infiltration in lung tissues, a response significantly more pronounced than that induced by purified endotoxin [109]. In Gram-positive pathogens, *S. aureus* BEVs exhibit broad cytotoxicity against host cells, delivering cholesterol-dependent virulence factors that induce cell death [110,111]. Notably, vesicle-associated α-hemolysin from *S. aureus* exhibits enhanced cytotoxic activity toward HaCaT human keratinocytes compared to its soluble form, mirroring observations in Gram-negative bacteria and mammalian exosomes [110,112]. This potency is hypothesized to arise from three mechanisms: (1) concentrated cargo delivery via membrane fusion, which avoids diffusion-driven dilution; (2) protection from immune clearance by antibodies and proteases; and (3) direct intracellular toxin translocation. Unlike soluble toxins that act extracellularly, vesicle-encapsulated toxins are delivered into the host cytosol through endocytosis or membrane fusion, enabling organelle disruption and membrane permeabilization [113]. The pathogenic potential of BEVs is further underscored by their diverse virulence payloads. For instance, *Fusobacterium nucleatum*-derived BEVs carry factors that polarize macrophages toward an M1 phenotype and disrupt intestinal epithelial barriers via FADD-RIPK1-caspase 3-mediated necroptosis, facilitating bacterial invasion [114]. Additionally, *P. aeruginosa* BEVs deliver a cytotoxic arsenal, including Cif protease, alkaline phosphatase, and PlcH phospholipase, to induce damage in human bronchial epithelial cells [54].

BEVs, which are commonly found in various ecosystems and environmental niches, including atmospheric dust [79,115], seawater [80], fecal matrices [81], and soil microenvironments [82], serve as effective vectors for the horizontal dissemination of virulence determinants. Their presence in these environments facilitates the cross-compartmental transfer of pathogenic materials, thereby intensifying microbial contamination, disrupting ecological balance, and posing significant risks to human health through both direct and indirect exposure pathways.

### 3.3. BEVs as Environmental Markers

Bioactive molecular cargo carried by BEVs has the potential to modulate the function of target cells, thereby influencing immune responses related to asthma and chronic obstructive pulmonary disease. Lung epithelial cell-derived extracellular vesicles (EVs) facilitate intercellular signaling and miRNA exchange. Clinical studies have reported elevated circulating EV-specific IgG antibody titers in the sera of patients compared to healthy controls [116]. These findings highlight the potential of BEVs as diagnostic biomarkers for respiratory diseases, with serum antibody providing a non-invasive strategy for assessing lung disease risk [117]. The biomedical applications of BEVs capitalize on their natural targeting properties and biocompatibility. For instance, Liu et al. [118] demonstrated that engineered BEVs can penetrate the osteoporotic bone microenvironment, accumulating in trabecular tissue to promote osteoblast differentiation while suppressing osteoclastogenesis. Additionally, labeling OMVs from *Akkermansia muciniphila* with the lipophilic dye DiR iodide allowed for in vivo tracking, revealing their role in regulating bone metabolism. Quantifying these OMVs could theoretically serve as a surrogate marker for evaluating the efficacy of *A. muciniphila*-mediated therapies in correcting skeletal dyshomeostasis. From an environmental health perspective, LPS are recognized as persistent contaminants in drinking water systems [119,120]. Detectable in source waters and treated effluents, LPS primarily originate from bacterial cell envelopes, including those released as BEVs [121,122]. The relationship between BEVs and LPS in aquatic environments underscores the utility of BEV quantification as a complementary indicator of water quality, especially considering that nano-sized BEVs may evade conventional water treatment processes. This necessitates the development of targeted removal strategies to mitigate health risks associated with endotoxins.

**Figure 4:**
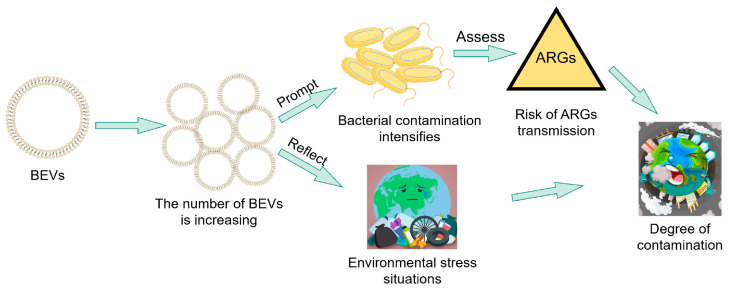
Schematic diagram of BEVs as potential biomarkers for microbial contamination. Elevated BEV abundance serves as a key indicator of bacterial contamination. It can be used to assess the environmental dissemination risk of ARGs and reflects overall microbial stress levels, providing a valuable metric for monitoring microbial pollution.

Despite their emerging significance, the utility of BEVs as environmental biomarkers remains largely underexplored. These nanovesicles serve as proxies for microbial distribution and metabolic activity within ecosystems, with elevated BEV abundances in water bodies or soils often correlating with increased levels of bacterial contamination. In addition to reflecting microbial load, BEVs encapsulate ARGs and mobile genetic elements, rendering them critical indicators for assessing the risks of ARG dissemination in the environment and predicting the evolution of antimicrobial resistance within microbial communities. Furthermore, the production of BEVs is closely linked to bacterial stress responses; under environmental perturbations, particularly antibiotic exposure, bacteria upregulate vesicle secretion as an adaptive strategy. Analyzing the abundance, composition, and cargo profiles of BEVs can elucidate the nature and intensity of stressors affecting microbial populations, thereby providing mechanistic insights into changes in environmental quality and associated ecological risks (Figure 4). However, the translation of these biomarkers into practical tools is impeded by technical limitations in the isolation and characterization of BEVs. Environmental matrices present unique challenges: large-volume samples, such as seawater and soil extracts, necessitate robust preprocessing, while complex mixtures of non-vesicular entities, including phage particles, cell debris, and soluble macromolecules, complicate purity. Size-based separation techniques, such as ultracentrifugation or nanoparticle tracking analysis, struggle to differentiate BEVs from viruses (20–200 nm), as both share overlapping size distributions. Current methodologies, including density gradient centrifugation and size-exclusion chromatography, achieve only partial enrichment and are unable to eliminate viral contaminants, particularly in samples with high phage titers. These limitations highlight the need for the development of vesicle-specific markers, such as membrane protein signatures, or the implementation of single-vesicle flow cytometry technologies to address the interference between BEVs and viruses. This is essential for ensuring accurate biomarker validation in complex environmental settings. Given the existing technological bottlenecks, a proposed direction for biomarker development involves the creation of antibody-conjugated magnetic beads that target BEV-specific membrane proteins, such as OmpA in Gram-negative bacteria and Sortase A in Gram-positive bacteria. This approach aims to facilitate the rapid enrichment and quantification of BEVs in complex matrices.

## 4. Discussion

Current methodologies for isolating and characterizing BEVs are hindered by the absence of efficient, target-specific separation protocols [42]. Traditional techniques, such as differential centrifugation and density gradient centrifugation, often yield insufficient purity due to the co-isolation of non-vesicular debris, including phage particles and cell fragments. Additionally, methods like nanoparticle tracking analysis and flow cytometry encounter difficulties in differentiating BEVs from structurally similar extracellular nanoparticles within complex environmental matrices [123,124]. These challenges are exacerbated by the lack of universal molecular markers for BEV identification, as assay accuracy is frequently compromised by background interference from soluble macromolecules and microbial byproducts [83,124]. Therefore, there is an urgent need to establish standardized and quality-controlled methods for BEV extraction and separation to ensure reproducibility and comparability across studies. Our mechanistic understanding of BEV biogenesis across diverse bacterial taxa remains fragmented [42]. Although the fundamental pathways of vesicle formation, such as membrane blebbing and explosive lysis, have been described in model species, the regulatory networks that govern BEV production under complex environmental conditions are still poorly characterized [42]. Furthermore, strain-specific variations in vesicle composition and release kinetics complicate the development of generalizable predictions, highlighting the need for comparative studies across bacterial genera to elucidate the genetic and environmental drivers of BEV biogenesis [125]. The environmental fate and ecological interactions of BEVs represent an underexplored frontier, with limited research addressing their transport dynamics in heterogeneous media, such as soil pore space [82], or their stability under varying abiotic conditions, including pH [47] and temperature [51]. Studies have demonstrated a significant increase in the quantity of BEVs in cadmium-contaminated soils [126], BEVs secreted by *Helicobacter pylori* can serve as a mechanism for bismuth extrusion [127]. However, critical knowledge gaps remain regarding the interactions of BEVs with co-existing pollutants, such as heavy metals, organic contaminants, and engineered nanomaterials, and how these interactions influence vesicle-mediated gene transfer, toxin delivery, and the structuring of microbial communities. Most research has concentrated on short-term exposures in controlled laboratory environments, leaving the long-term effects of BEV accumulation, including transgenerational impacts on microbial evolution and ecosystem resilience, largely unexplored. From both technological and regulatory perspectives, there is a lack of targeted strategies for environmental monitoring and pollution mitigation focused on BEVs [128]. Current risk assessment frameworks overlook the unique properties of these nanovesicles, such as their ability for long-range transport and persistence, while remediation technologies, including membrane filtration and advanced oxidation processes, have not been optimized for BEV removal [128]. Developing integrative approaches that combine single-vesicle analytics, multiscale ecological modeling, and engineered barrier systems will be crucial to address these knowledge and technological gaps, thus facilitating evidence-based management of BEV-associated contamination risks.

Future investigations should prioritize the development of next-generation analytical methodologies to address the critical need for sensitive and specific detection of BEVs in complex environmental matrices. By leveraging advancements in nanomaterial science and biosensor technology, researchers can design affinity-based capture systems, such as antibody-conjugated magnetic beads, or label-free sensing platforms and surface plasmon resonance devices to overcome the limitations of current techniques. These innovations would enable single-vesicle resolution and multiplexed cargo profiling, facilitating accurate quantification of BEVs amidst interfering nanoparticles, including viruses and cell debris, and unlocking their potential as robust biomarkers for microbial contamination. Furthermore, elucidating the molecular mechanisms of BEV biogenesis represents another fundamental research frontier. Current knowledge of vesicle formation, which encompasses intracellular cargo sorting, membrane curvature modulation, and exocytotic release, remains fragmented across different bacterial taxa. Future studies should integrate omics approaches, such as transcriptomics and proteomics, with single-cell imaging to decode the genetic regulatory networks and post-translational modifications that govern BEV production under diverse environmental conditions, including nutrient stress, oxidative damage, or antibiotic exposure. Comparative analyses across bacterial phyla will be critical in identifying conserved biogenesis pathways versus strain-specific adaptations, providing mechanistic insights into how microbes tune vesicle output in response to environmental cues. Additionally, the role of BEVs in pollutant transport and transformation warrants systematic investigation, particularly regarding their capacity to mediate the horizontal transfer of contaminants, such as heavy metals, microplastics, and persistent organic pollutants, across ecological interfaces. Mechanistic investigations should elucidate the interactions between BEVs and environmental pollutants, focusing on adsorption kinetics, cargo encapsulation efficiency, and interphase transport dynamics across aquatic, terrestrial, and atmospheric systems. BEVs can enhance the mobility of heavy metals through chelation and promote the biodegradation of plastic particulates through enzyme-enriched vesicular cargos, offering new opportunities for biologically mediated pollutant remediation. By utilizing their nanoscale structure, membrane stability, and biodegradable composition, BEVs hold significant potential for the development of advanced bioremediation technologies. Progress in genetic engineering further enables the design of bacterial strains that secrete BEVs containing pollutant-degrading enzymes such as laccases for the oxidative breakdown of organic contaminants, or metal-binding peptides for selective heavy metal capture. These engineered vesicles can serve as highly specific biocatalysts for pollutant degradation. In addition, BEV-inspired nanomaterials with customized surface properties may enhance conventional remediation methods such as membrane filtration and advanced oxidation processes by improving pollutant capture efficiency and reducing ecological toxicity. When combined with real-time environmental monitoring systems that employ BEV-derived biomarkers, these approaches could fundamentally transform environmental management by addressing both the sources and ecological consequences of BEV-mediated pollution.

## 5. Conclusions

Bacterial extracellular vesicles (BEVs) are emerging bio-nanoparticles that play multifaceted roles in environmental systems by mediating the dissemination of antibiotic resistance genes, virulence factors, and pollutants. Their nanoscale stability and ubiquity across diverse ecosystems make them both potent agents of microbial adaptation and promising bioindicators of environmental stress. However, challenges remain in elucidating BEV–pollutant interactions, achieving field-level quantification, and developing efficient removal or detection technologies due to their structural similarity to viruses and other nanoparticles. Future research should focus on clarifying the mechanisms governing BEV formation and pollutant association, advancing sensitive and specific analytical tools, and designing engineered vesicles or BEV-inspired nanomaterials for targeted pollutant degradation. These efforts will be crucial for integrating BEV dynamics into environmental monitoring and remediation frameworks, thereby enhancing ecological resilience and public health protection.

## Data Availability

No new data were created or analyzed in this study. Data sharing is not applicable to this article.
